# Imaging of antitubercular dimeric boronic acids at the mycobacterial cell surface by click-probe capture†

**DOI:** 10.1039/d2cc02407a

**Published:** 2022-08-02

**Authors:** Collette S. Guy, Ruben M. F. Tomás, Qiao Tang, Matthew I. Gibson, Elizabeth Fullam

**Affiliations:** School of Life Sciences, University of Warwick Coventry CV4 7AL UK e.fullam@warwick.ac.uk; Department of Chemistry, University of Warwick Coventry CV4 7AL UK; Division of Biomedical Sciences, Warwick Medical School, University of Warwick Coventry CV4 7AL UK

## Abstract

Dimeric boronic acids kill *Mycobacterium tuberculosis* (*Mtb*) by targeting mycobacterial specific extracellular glycans, removing the requirement for a therapeutic agent to permeate the complex cell envelope. Here we report the successful development and use of new ‘clickable’ boronic acid probes as a powerful method to enable the direct detection and visualisation of this unique class of cell-surface targeting antitubercular agents.

## Abstract 2

Antitubercular ‘clickable’ diboronic acid agents are directly incorporated into the mycobacterial cell envelope through glycan-targeting.

## Graphical abstract

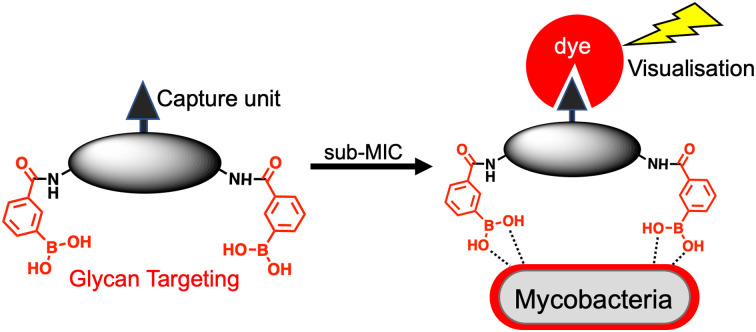

*Mycobacterium tuberculosis* (*Mtb*) is one of the world's most successful human pathogens. Tuberculosis (TB) kills more people than any other bacterial infectious agent each year, with over 1.5 million deaths from TB and ∼10 million new TB cases reported by the World Health Organisation in 2020.^1^ As a result of the COVID-19 pandemic TB mortality rates are increasing for the first time in ∼15 years, which has set back efforts in the global management of TB.^2^ Although the current antibiotic regimen is effective in eliminating drug-susceptible *Mtb* infections, resistance to front-line agents is increasingly common reducing successful treatment outcomes.^1,3,4^ Therefore, to tackle this global health threat we urgently need new antitubercular therapeutics equipped with novel mechanisms of action, alongside improved diagnostics.

A substantial bottleneck in the development of new TB drugs is the highly impermeable mycobacterial cell envelope, which acts as an intrinsic barrier preventing many molecules, including antibiotics, from accessing the cytoplasm.^5–7^ To avoid the requirement for an antitubercular agent to cross the mycobacterial cell envelope, we have recently shown that pathogen specific killing of *Mtb* can be achieved by exploiting glycan-targeting boronic acid and boroxole agents that engage with mycobacterial specific cell-surface glycans not present in other bacterial species and mammalian cells.^8,9^ We reported that the extracellular targeting mechanism requires the multimeric display of the glycan-targeting units^8,9^ and is distinct from cell-permeable monomeric boronic acids, which target β-lactamase and penicillin binding proteins.^8,10–12^ A key design feature was the spacing between the boronic acid and boroxole moieties. In this instance, a short linker between two boron containing moieties was crucial for antimycobacterial activity compared to long poly(ethylene glycol) scaffolds (Fig. 1A).^8,9^ The resulting dimeric boronic and boroxole analogues displayed selective interactions with isolated *Mtb* cell envelope constituents containing glycans with *cis*-diols.^8,9^ Both analogues facilitated the capture of the trehalose containing glycolipid: trehalose dimycolate. In contrast, the dimeric boronic acid engaged with a wider array of cell envelope components, including trehalose monomycolate, lipomannan, lipoarabinomannan, arabinogalactan and peptidoglycan.^8,9^ An important implication of this glycan-targeting concept is that a new class of antitubercular agents can be developed with specific recognition motifs designed to interact directly with key components of the mycobacterial cell envelope to kill *Mtb*. This is a major advance in the quest for novel TB drugs since this strategy bypasses the requirement for a therapeutic agent to cross the ‘waxy’ *Mtb* cell envelope, and is distinct from conventional approaches. Indeed, glycan-targeting antibodies have reached the clinic, highlighting the potential of this approach.^13^

**Fig. 1:**
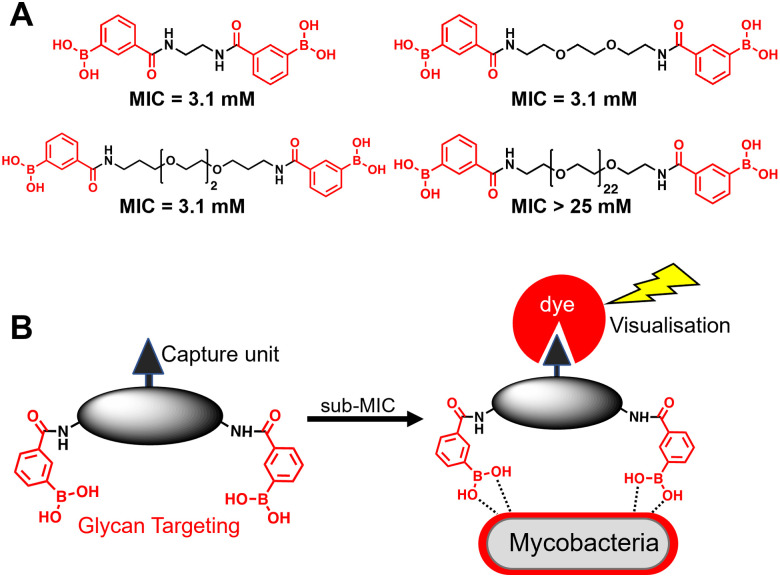
(A) Dimeric boronic acids and MIC values previously reported against *Mycobacterium smegmatis*.^8^ (B) Strategy for ‘click’-capture and analysis of dimeric boronic acid engagement with the mycobacterial cell envelope.

In this work, we sought to demonstrate the interaction of dimeric boronic acids with the mycobacterial cell envelope through direct visualisation *via* a click-based fluorescence strategy (Fig. 1B). We designed and synthesised two new dimeric boronic acid derivatives bearing either an azido- or alkynyl-handle for post-mycobacterial engagement labelling. The compounds retain antimycobacterial activity following incorporation of the azido and alkyne modifications into the scaffold and provide direct evidence that the dimeric boronic acids are localised to the mycobacterial cell surface. This confirms that extracellular therapeutics or, potentially, diagnostics can be developed using this platform.

Based on our previous findings,^8^ we rationalised that incorporation of ‘clickable’ capture units into the middle of the linker unit whilst retaining the essential spacing between the two 3-carboxy-phenyl boronic acids to mimic the structure of our most potent dimeric boronic acids^8^ would not interfere with antimycobacterial potency. Our approach to add the fluorogenic probe in the second-step, rather than incorporating this functionality directly into the dimeric boronic acid scaffold, is crucial since fluorophore reporter dyes, which tend to be hydrophobic, could promote non-specific interactions or engagement with the mycobacterial cell envelope.^14^

The synthetic route to the new probes containing an azido or alkyne group is shown in Scheme 1 and Scheme S1 (ESI†). B2-alkyne and B2-N_3_ were prepared from commercial bis-MPA (2,2-bis(hydroxymethyl)propionic acid) cores containing either an azide or alkyne unit, which were used as the scaffolds to install both 3-carboxy-phenyl boronic acid units in two steps and in ∼30% yield. First the *N*-Boc bis-MPA precursors were deprotected with trifluoroacetic acid followed by an acyl-chloride coupling promoted by triethylamine to install the 3-carboxy-phenyl boronic acid groups in one-step, giving access to the B2-alkyne and B2-N_3_ dimeric boronic acid probes (see ESI† for full experimental and characterisation data).

**Scheme 1:**
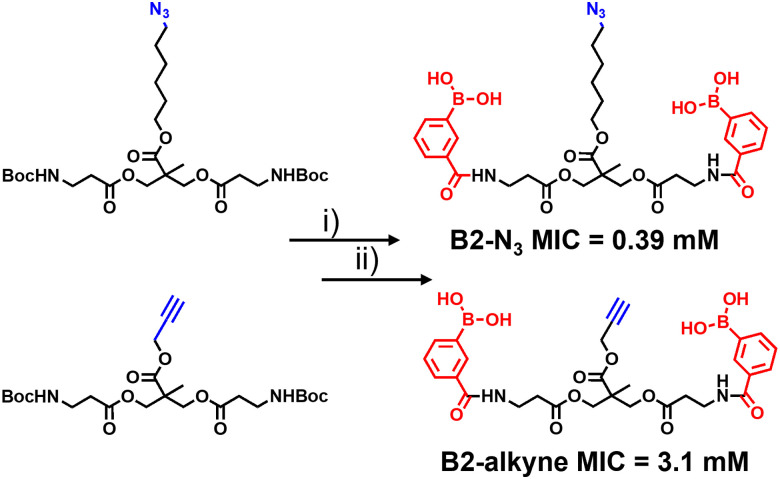
Synthesis of antimycobacterial ‘clickable’ dimeric boronic acids. (i) TFA, 3 h rt. (ii) 3 eq. 3-(chlorocarbonyl)phenyl boronic acid, 6 eq. Et_3_N, DCM, 0 °C 30 min, rt 16 h. Minimum inhibitory concentration (MIC) against *Mycobacterium smegmatis*.

With the two boronic acid probes (B2-alkyne and B2-N_3_) in hand, our first objective was to determine whether the addition of the azido or alkyne modifications impacted on antimycobacterial potency. Evaluation of the minimum inhibitor concentrations (MICs) using the resazurin reduction assay^15^ found that both compounds killed *Mycobacterium smegmatis* with similar MIC values to those reported using related dimeric boronic acids with a PEG-based linker (Fig. 1 and Scheme 1),^8,9^ indicating that the clickable handles do not interfere with function. Interestingly, the azido derivative was more potent than the alkyne derivative with a MIC of 0.39 mM compared to 3.1 mM for the azido derivative (Scheme 1).

The first approach to directly visualise the interaction between the dimeric boronic acids with mycobacteria was with B2-alkyne and the fluorogenic azide probe CalFluor 488 azide (Az488), Fig. 2A. Az488 is only fluorescent following the reaction with an alkyne *via* Cu-catalysed alkyne–azide [3+2] cycloaddition (CuAAc).^16^ A potential advantage of this approach is that no wash steps are required after the fluorescence generating CuAAc reaction to remove unreacted probe, which may be preferred for either low affinity, transient or non-covalent interactions. *M. smegmatis*, as a fast-growing non-pathogenic model system of *Mtb*, were cultured to logarithmic phase and exposed to sub-MIC of B2-alkyne (1 mM) for 15 min followed by incubation with phenylboronic acid (2 mM) lacking the alkyne targeting moiety to compete with the B2-alkyne. Under these assay conditions, no killing of mycobacteria was observed. The cells were fixed before a second labelling step with Az488 and imaged directly, Fig. 2B. Additionally, control cells were incubated in the absence of B2-alkyne and exposed to unlabelled phenylboronic acid and the same Cu-‘click’ labelling procedure. Fluorescence microscopy revealed cell surface labelling of the mycobacterial cells treated with the B2-alkyne compared to control cells (Fig. 2B), indicating that the dimeric boronic acid B2-alkyne is readily incorporated into the mycobacterial cell envelope. We were not able to quantify Az488 labelled mycobacterial cells with flow cytometry analysis although a labelled sub-population was present (Fig. S3, ESI†), perhaps suggesting that non-covalent boronic acid interactions coupled with flow cytometry dilution effects leads to the release of the boronic acid from the cell envelope.

**Fig. 2:**
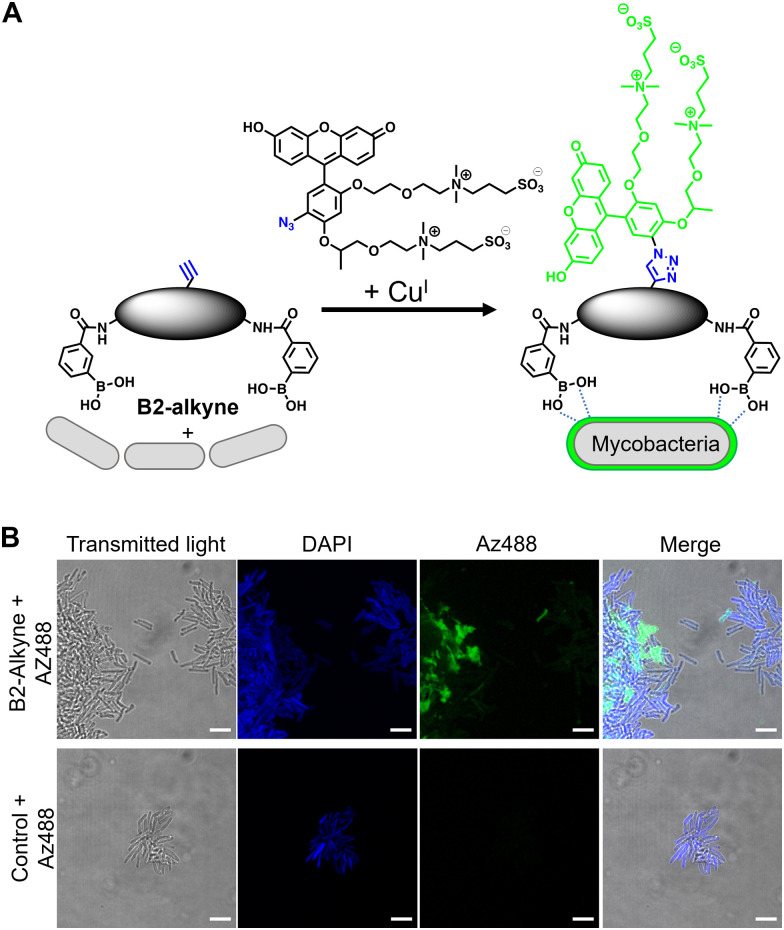
(A) Strategy for ‘click’-capture of B2-alkyne with fluorogenic CalFluor 488 azide. (B) Fluorescence microscopy of B2-alkyne labelled *Mycobacterium smegmatis* with Az488 *via* CuAAc. Scale bars are 5 μm.

In order to investigate the interaction of dimeric boronic acids on live mycobacterial cells, next we evaluated the more potent B2-N_3_ reporter with the brighter DBCO-Cy3 dye (Fig. 3A). *M. smegmatis* cells were cultured to mid-logarithmic phase and in this instance exposed to sub-MIC levels of B2-N_3_ (100 μM) for 30 min. After this time the cells were washed to remove any free B2-N_3_ and then exposed to the DBCO-Cy3 secondary label to enable strain promoted Cu-free azide-alkyne cycloaddition (SPAAC) reaction of DBCO-Cy3 with B2-N_3_.^17^ The cells were washed to remove unreacted DBCO-Cy3 and analysed by microscopy (Fig. 3B) and flow cytometry. As described before, untreated cells exposed to DBCO-Cy3 in the absence of B2-N_3_ and untreated mycobacterial cells were also evaluated. Interestingly, the majority of *M. smegmatis* cells incubated with B2-N_3_ showed fluorescent labelling (Fig. 3B), with a higher efficiency than B2-alkyne and, as expected, no background fluorescence was observed for the control cells. Super-resolution live cell imaging with an Airyscan detector^18^ found the fluorescent signal was predominantly localised at the cell surface (Fig. 3B), confirming the interaction of B2-N_3_ with components of the mycobacterial cell envelope. The higher resolution imaging of B2-N_3_ can be ascribed to a combination of increased antimycobacterial potency of B2-N_3_, suggesting stronger interactions with the cell envelope and the higher fluorescence quantum yield of DBCO-Cy3 (0.15)^19^ compared to CalFluor Az488-triazole conjugate (0.07).^16^ Similar to B2-alkyne, we were unable to quantify the extent of B2-N_3_ mycobacterial labelling with flow cytometry but also observed a sub-population of fluorescently labelled cells (Fig. S4, ESI†), supporting that boronic acids have a pivotal role in modifying the mycobacterial cell surface.

**Fig. 3:**
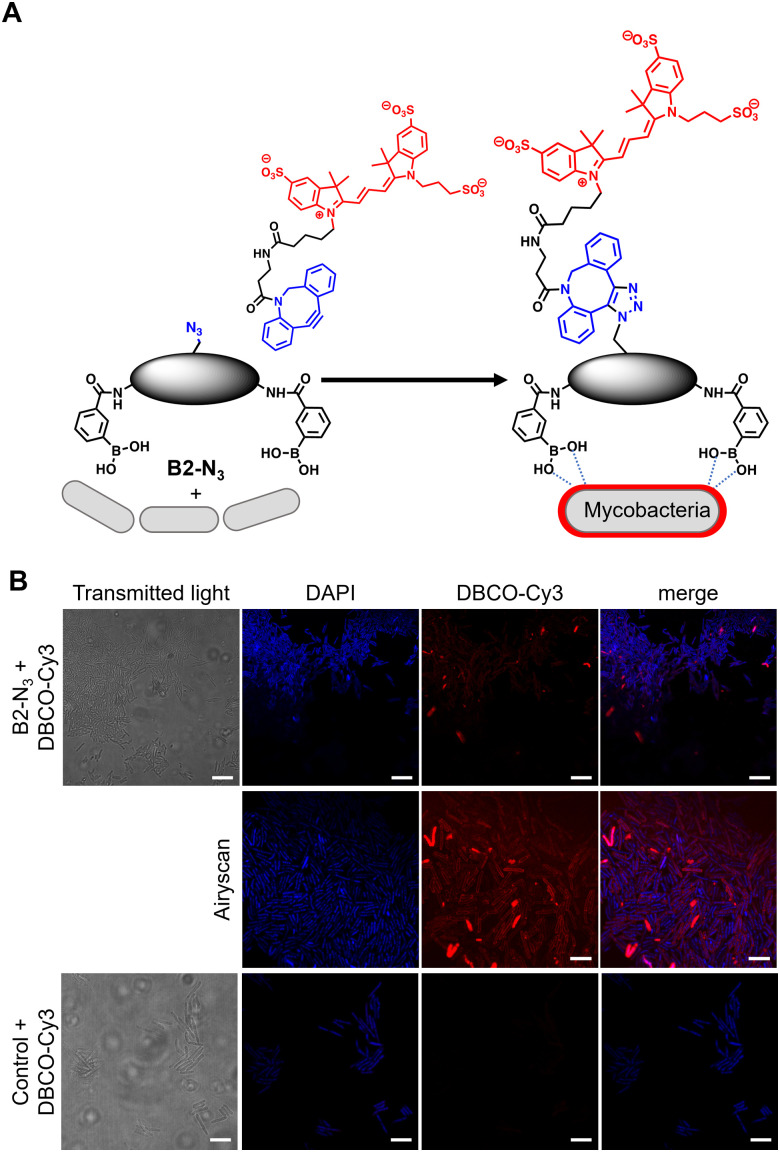
(A) Strategy for ‘click’-capture of B2-N_3_ with DBCO-Cy3 (B) fluorescence microscopy of B2-N_3_ labelled *Mycobacterium smegmatis* with DBCO-Cy3 *via* SPAAC. Scale bars are 5 μm.

In conclusion, we have developed a strategy to provide direct evidence that antitubercular dimeric boronic acids target extracellular mycobacterial cell surface components. This was achieved through the rational design and synthesis of new modular dimeric boronic acids with precision spacing between the glycan-chelating boronic acid moieties and a ‘clickable’-recruitment handle in the centre of the linker unit. A pro-fluorescence ‘click’ strategy revealed binding but did not provide sufficient resolution. In contrast, copper-free click recruitment of DBCO-Cy3 on live mycobacterial cells revealed substitutional cell labelling as shown by microscopy. Importantly, this two-step ‘click’ capture approach confirms that dimeric boronic acids are retained on the exterior of the mycobacterial cells, thus demonstrating modification the cell envelope. Taken together, these results validate that new drugs, and diagnostics, for mycobacteria can be discovered by targeting the unique extracellular glycans rather than the traditional approaches of targeting specific intracellular pathways, which often fail due to the low permeability of mycobacteria. It is our hope that this approach will lead to the development of novel TB therapeutics alongside new imaging and diagnostic platforms.

This work was supported by a Sir Henry Dale Fellowship to EF jointly funded by the Wellcome Trust and Royal Society (104193/Z/14/Z and 104193/Z/14/B), research grants from the Royal Society (RG120405) and the Leverhulme Trust (RPG-2019-087) and the EPSRC MAS doctoral training centre for a studentship to RMFT (EP/L015307). We thank Ian Hands-Portman for technical assistance with the microscopy studies. For the purpose of open access, the author has applied a Creative Commons Attribution (CC BY) licence to any Author Accepted Manuscript version arising from this submission.

## Conflicts of interest

There are no conflicts to declare.

## Supplementary Material

CC-058-D2CC02407A-s001
