# Supplementary material for: Imaging of antitubercular dimeric boronic acids at the mycobacterial cell surface by click-probe capture
Source: Chem Commun (Camb). 2022 Aug 2;58(67):9361–4. doi: 10.1039/d2cc02407a (PMC9387567; doi:10.1039/d2cc02407a)
Supplement: CC-058-D2CC02407A-s001 [file CC-058-D2CC02407A-s001.pdf]

Electronic Supplementary Material (ESI) for ChemComm.
This journal is © The Royal Society of Chemistry 2022

Supporting Information

Imaging of antitubercular dimeric boronic acids at the mycobacterial cell surface by click-probe capture

Collette S Guy,[‡a] Ruben M. F. Tomás,[‡bc] Qiao Tang,[b] Matthew I Gibson,[bc] Elizabeth Fullam,[*a]

[a] School of Life Sciences, University of Warwick, Coventry, CV4 7AL, UK.
E-mail: e.fullam@warwick.ac.uk
[b] Department of Chemistry, University of Warwick, Coventry, CV4 7AL, UK
[c] Warwick Medical School, University of Warwick, Coventry, CV4 7AL, UK
[‡]These authors contributed equally to this study

Table of Contents

**Supplementary Results**	S3
Scheme S1 Synthetic route for the synthesis of 3-carboxy-phenyl boronic acid bis-MPA dendrons	S3
Fig. S1. Fluorescence scans of B2-alkyne and CalFluor Azide 488	S4
Fig. S2. Fluorescence scans of B2-N3 and DBCO-Cy3	S5
Fig. S3 Flow cytometry analysis of Mycobacterium smegmatis cells treated with B2-alkyne and labelled with CalFluor Azide 488	S6
Fig. S4 Flow cytometry analysis of *Mycobacterium smegmatis* cells treated with B2-N_3 and labelled with DBCO-Cy3	S7
**Supplementary Chemical and Synthetic protocols**	S8
**General Information and Procedures**	S8
**Synthetic Procedures**	S9
Polyester bis-MPA dendron (2 phenyl boronic acid, 1 acetylene(core), generation 1) (**5**)	S9
Fig. S5 ^{1}H NMR spectrum of B2-alkyne (**5**)	S10
Fig. S6 ^{13}C NMR spectrum of B2-alkyne (**5**)	S10
Polyester bis-MPA dendron (2 phenyl boronic acid, 1 azide(core), generation 1) **(8)**	S11
Fig. S7 ^{1}H NMR spectrum of B2-N_3 (**8**)	S12
Fig. S8 ^{13}C NMR spectrum of B2-N_3 (**8**)	S12
**Supplementary Biological protocols**	S13
Bacterial strains, culture conditions and chemicals	S13
Determination of minimum inhibitory concentrations	S13
Fluorescence analysis of B2-Alkyne and CalFluor Azide 488	S13
Fluorescence analysis of B2-N3 and DBCO-Cy3	S13
Labelling of *Mycobacterium smegmatis* with B2-alkyne and CalFluor Azide 488 dye for microscopy	S14
Labelling of *Mycobacterium smegmatis* with B2-alkyne and CalFluor Azide 488 dye for flow cytometry	S14
Labelling *Mycobacterium smegmatis* cells with B2-N_3 and DBCO-Cy3 dye for microscopy and flow cytometry	S14
Confocal microscopy	S15
Flow Cytometry	S15
**References**	S16

Supplementary Results

Scheme S1. Synthetic route for the synthesis of 3-carboxy-phenyl boronic acid bis-MPA dendrons

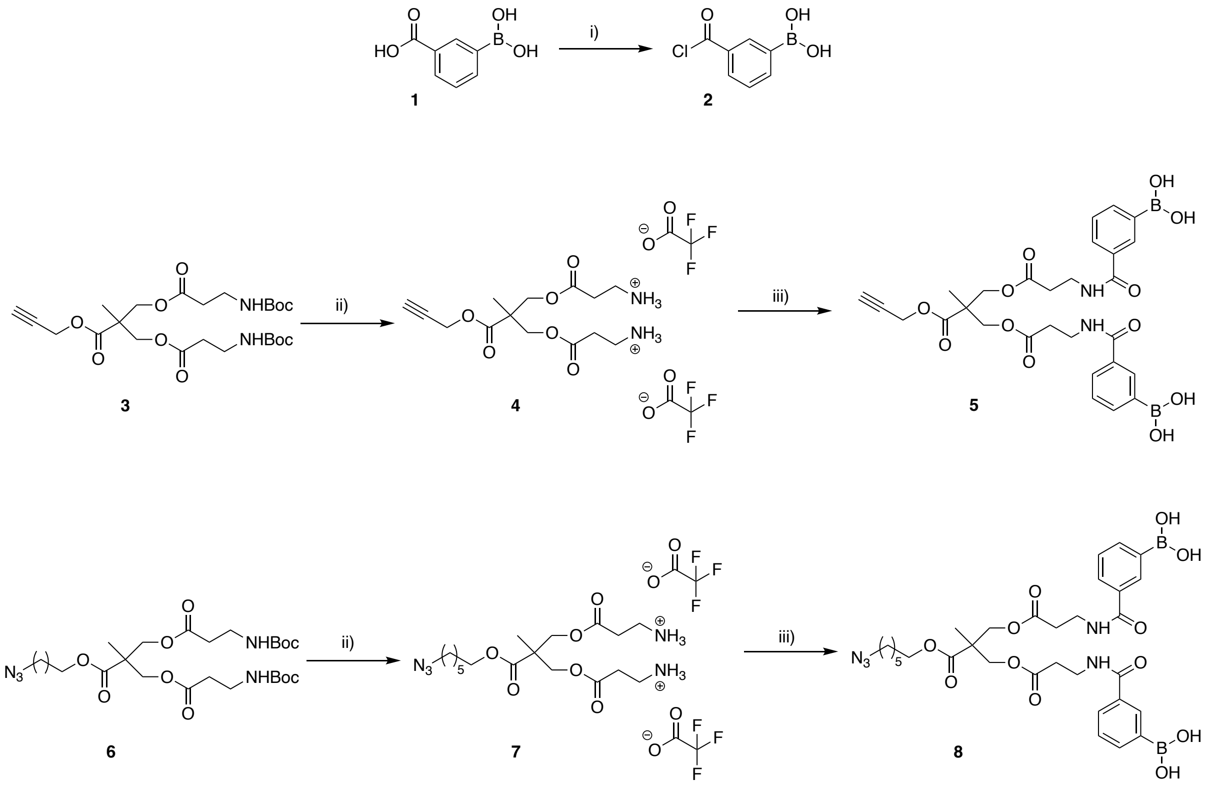

Reagents and conditions: i) 2.4 eq. oxalyl chloride, 0.2 eq. DMF, THF, 0 °C, 2h. ii) TFA, 3 h, rt. iii) 3 eq. 3-(chlorocarbonyl)phenyl boronic acid (**2**), 6 eq. Et_3N, DCM, 0 °C 30 min, rt 16 hr.

Fig. S1. Fluorescence scans of B2-alkyne and CalFluor Azide 488. Excitation wavelength: 488 nm and emission scan from 495 – 630 nm.

A) PBST,

B) PBST + B2-alkyne (1 mM),

C) PBST + CalFluor Azide 488 (10 µM),

D) PBST + CalFluor Azide 488 (10 µM) + $CuSO_4$ (50 µM) + bovine serum albumin (BSA) (0.2 mg/mL) + tris[(1-benzyl-1*H*-1,2,3-triazol-4-yl)methyl]amine (100 µM) + sodium ascorbate (2.5 mM),

E) PBST + B2-alkyne (1 mM) + Cal Fluor Azide 488 (10 µM) + $CuSO_4$ (50 µM) + BSA (0.2 mg/mL) + tris[(1-benzyl-1*H*-1,2,3-triazol-4-yl)methyl]amine (100 µM) + sodium ascorbate (2.5 mM).

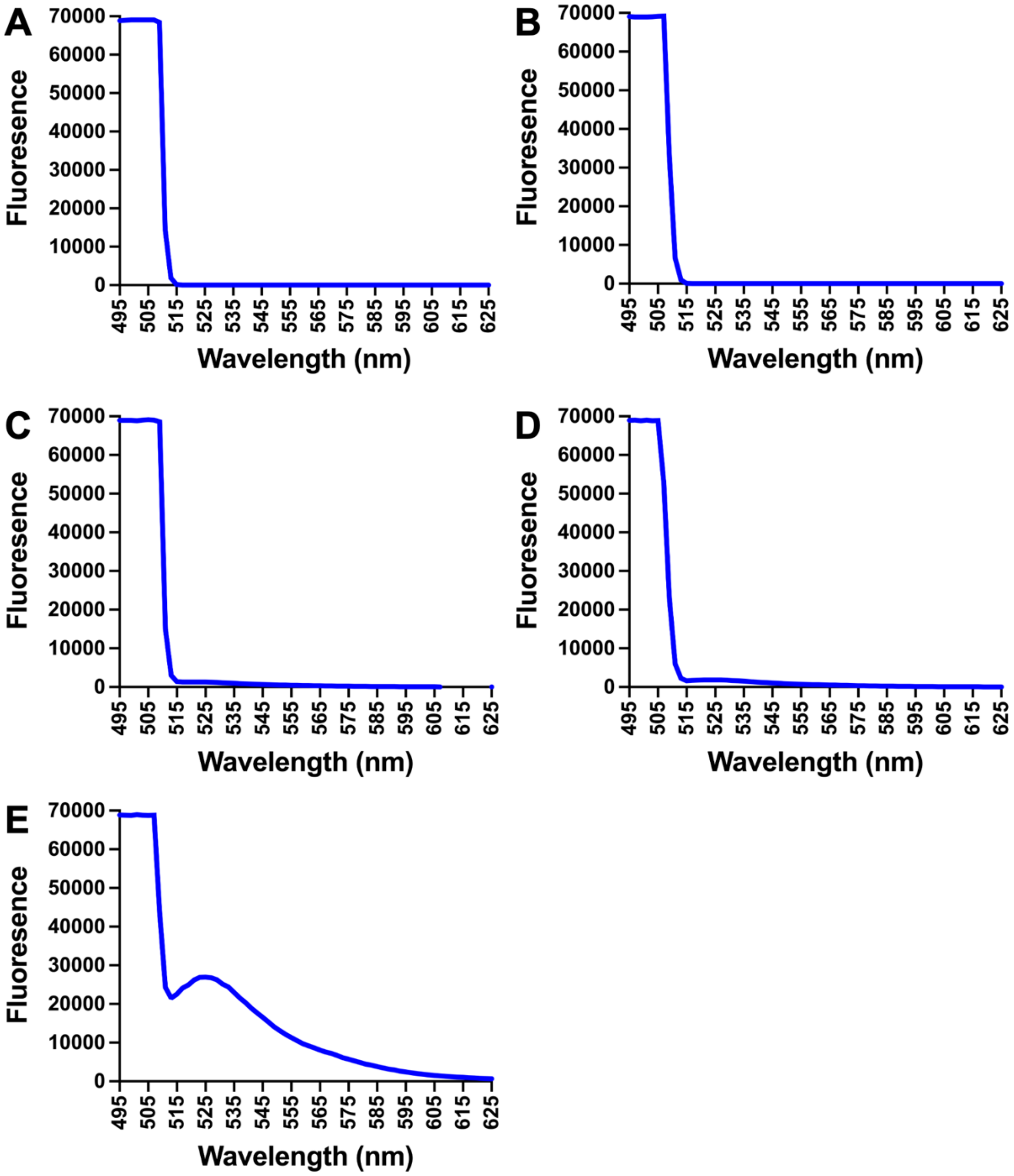

Fig. S2. Fluorescence scans of B2-N_3 and DBCO-Cy3. Excitation wavelength: 514 nm and emission scan from 538 – 680 nm.

A) PBST

B) PBST + DBCO-Cy3 (100 μM)

C) PBST + B2-N_3 (100 μM)

D) PBST + DBCO-Cy3 (100 μM) + B2-N (100 μM)

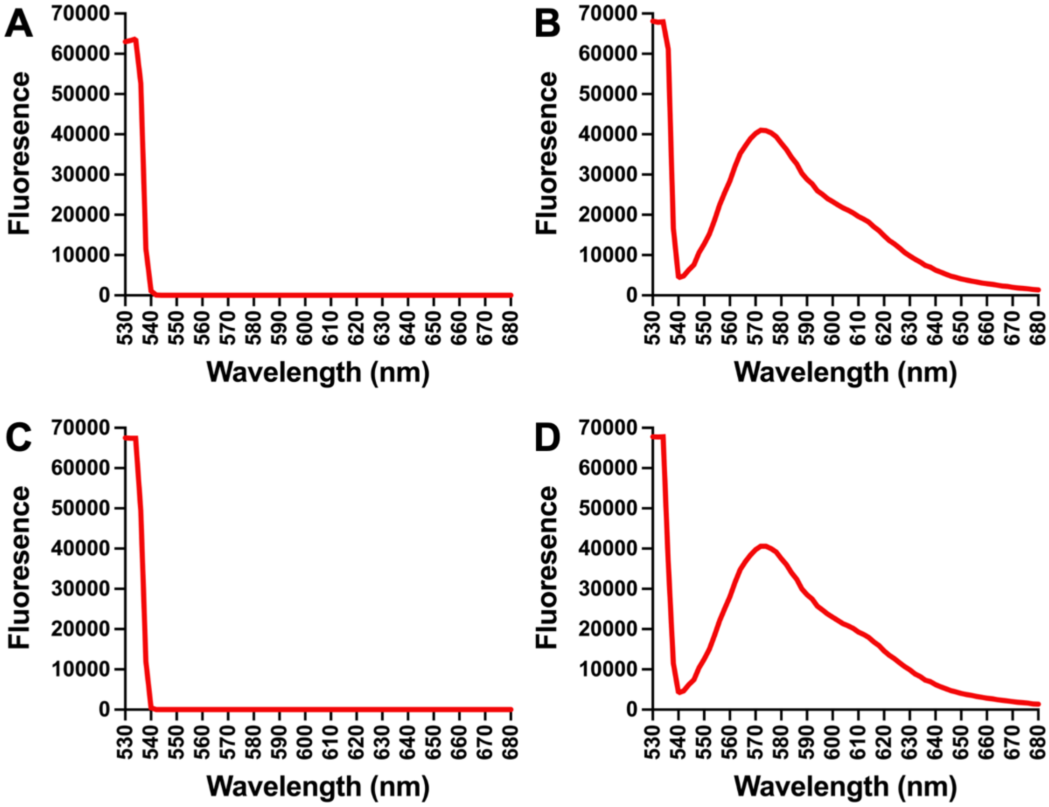

Fig. S3. Flow cytometry analysis of *Mycobacterium smegmatis* cells treated with B2-alkyne and then labelled with CalFluor Azide 488 *via* CuAAc. Number in red shows the geometric mean fluorescence of the cells A) Untreated cells, B) Cells treated with 0 mM B2-Alkyne then 10 µM CalFluor Azide 488, C) Cells treated with 1 mM B2-Alkyne then 10 µM Cal Fluor Azide 488, D) Overlay of the three samples. Untreated cells (red), cells treated with 0 mM B2-Alkyne then 10 µM CalFluor Azide 488 (green), cells treated with 1 mM B2-Alkyne then 10 µM Cal Fluor Azide 488 (blue).

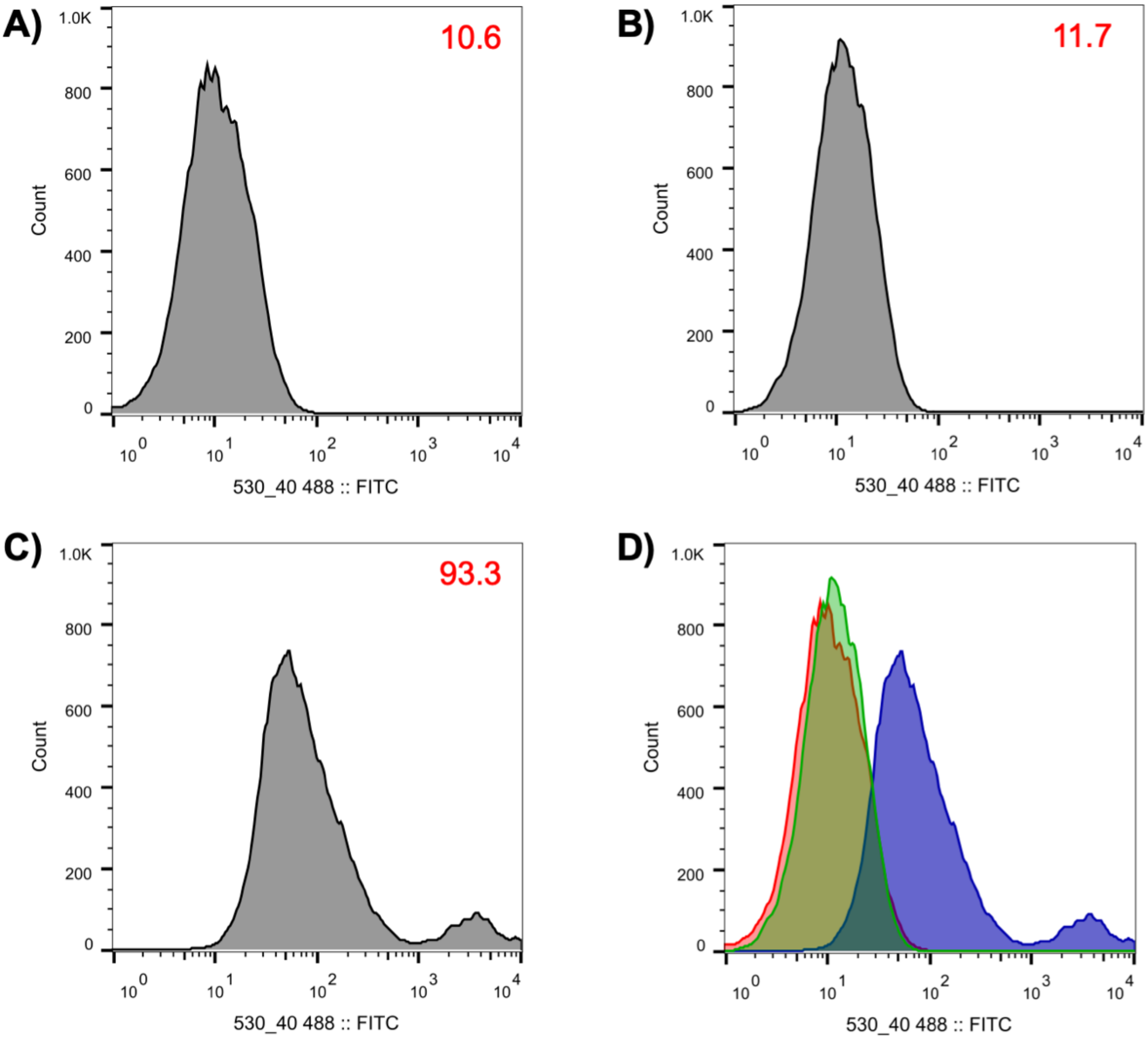

Fig. S4. Flow cytometry analysis of *Mycobacterium smegmatis* cells treated with B2-N_3 and then labelled with DBCO-Cy3 *via* SPAAC. Number in red shows the geometric mean fluorescence of the cells. A) Untreated cells, B) Cells treated with 0 µM B2-N_3 then 50 µM DBCO-Cy3, C) Cells treated with 50 µM B2-N_3 then 50 µM DBCO-Cy3, D) Overlay of the three samples. Untreated cells (red), cells treated with 0 mM B2-N_3 then 50 µM DBCO-Cy3 (green), cells treated with 50 µM B2-N_3 then 50 µM DBCO-Cy3 (blue)

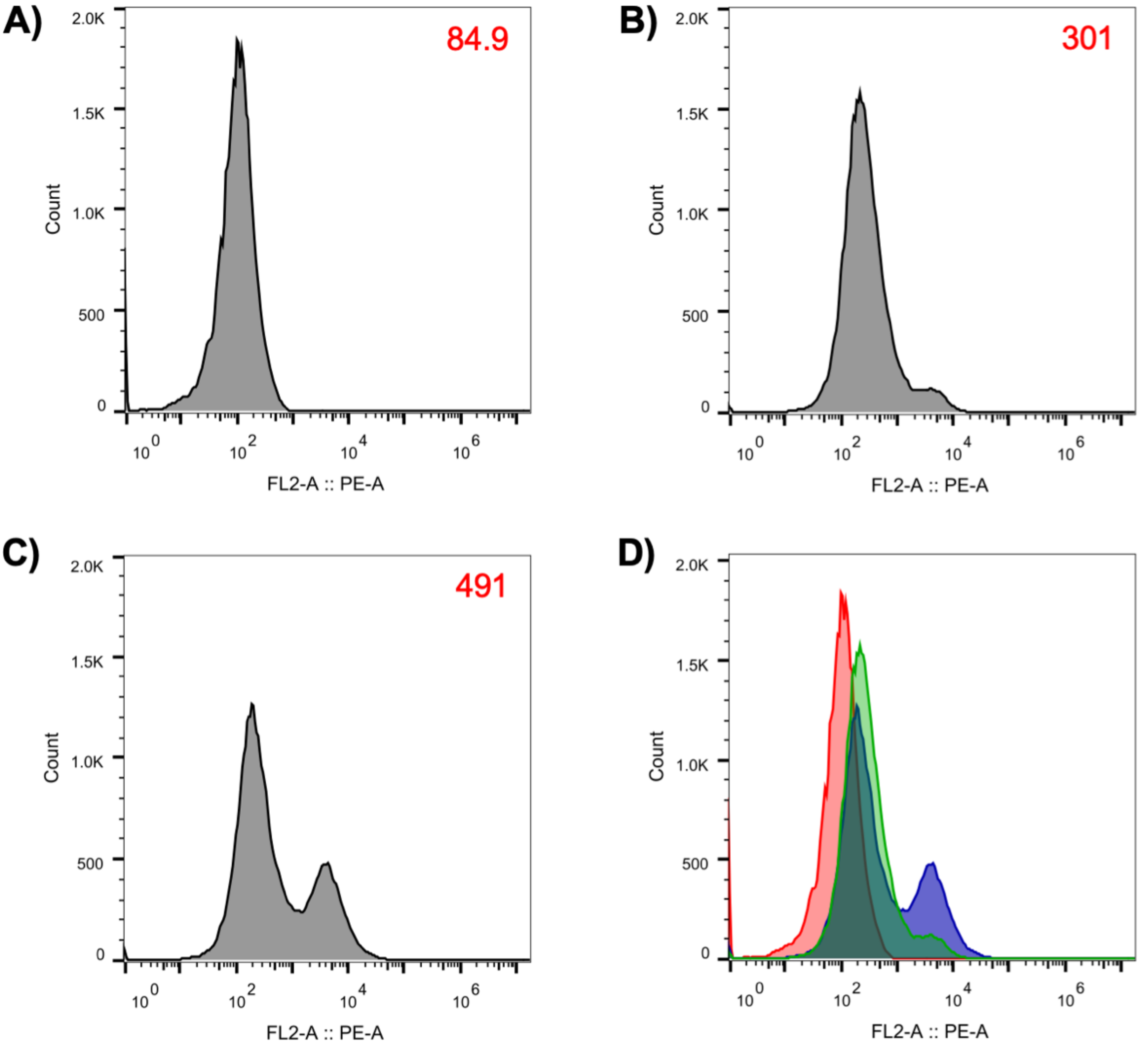

Supplementary Chemical and Synthetic protocols

General Information and Procedures

The chemicals and solvents, including anhydrous solvents, were used as supplied and without further purification. Polyester bis-MPA dendron (2 NHBoc, 1 azide(core), generation 1), Polyester bis-MPA dendron (2 NHBoc, 1 acetylene(core), generation 1), anhydrous tetrahydrofuran (THF) ≥99.9%, anhydrous N,N- dimethylformamide (DMF) ≥99.8%, anhydrous dichloromethane (DCM) ≥99.8% and oxalyl chloride 98% were purchased from Sigma-Aldrich. Methanol (MeOH), dichloromethane (DCM), triethylamine, ethyl acetate and isopropanol (iPrOH) were purchased from Fisher Scientific at laboratory reagent grade. 3-Carboxyphenylboronic acid 97% was purchased from Acros Organics. Deuteromethanol (MeOD) 99.8% was purchased from Apollo Scientific. Distilled water (H_2O) was used throughout.

All reactions were performed using oven dried. Thin Layer Chromatography (TLC) was carried out using Merck aluminium backed sheets coated with 60 F254 silica gel. Visualisation of the silica plates was achieved using a UV Lamp (λ = 254 nm), and/or potassium permanganate (1.5 g $KMnO_4$, 10 g KCO_3 and 1.25 mL 10% NaOH in 200 mL water) or Alizarin Red S (1 mM alizarin red S in acetone).[1] Flash chromatography was carried out using Merck silica gel 60, 35-75 μm as the stationary phase (Sigma Aldrich) unless otherwise stated. Mobile phases are reported in solvent ratios.

Proton (^{1}H) and carbon (^{13}H) NMR spectra were obtained at 298 K. ^{1}H NMR and ^{13}C DEPT NMR were recorded on a Bruker DPX-500 instrument. NMR were fully assigned using COSY, HSQC and HMBC. All chemical shifts are quoted in parts per million (ppm), using the residual solvent as the internal standard. (1H NMR: MeOD = 3.31, 13C NMR: MeOD = 49.0). Coupling constants (J) are reported in hertz (Hz) with the following abbreviations: s, singlet; d, doublet; t, triplet; q, quartet; quin, quintet; m, multiplet; br, broad.

Mass spectra were recorded on a Bruker HCT Ultra spectrometer using electrospray ionisation (ESI). M/z values are reported in Daltons.

Synthetic Procedures

Polyester bis-MPA dendron (2 phenyl boronic acid, 1 acetylene(core), generation 1) (5)

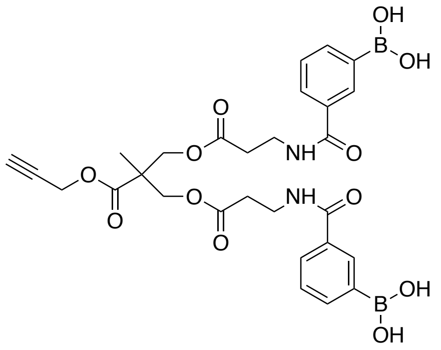

Polyester bis-MPA dendron (2 NHBoc, 1 acetylene(core), generation 1) (**3**) (50 mg, 0.095 mmol) was dissolved in trifluoroacetic acid (90 μL) and left at room temperature for 4 hours. The reaction was then concentrated *in vacuo* to give the deprotected amine (**4**) as a TFA salt. In a separate flask, 3-carboxyphenylboronic acid (48 mg, 0.291 mmol) was dissolved in anhydrous THF (3 mL) under nitrogen and cooled to 0 °C. DMF (4.5 μL, 0.058 mmol) was then added, followed by the dropwise addition of oxalyl chloride (59 μL, 0.699 mmol). The reaction mixture was then allowed to warm to room temperature and stirred for two hours before removing the solvent *in vacuo* to give crude 3-(chlorocarbonyl)phenyl boronic acid (**2**). The resulting residue was dissolved in anhydrous DCM (2 mL), under nitrogen, and cooled to 0 °C. A mixture of the deprotected TFA salt (**4**) (0.095 mmol) and triethylamine (81 μL, 0.582 mmol) in anhydrous DCM (1 mL) was then added dropwise and the resulting mixture was stirred at 0 °C for 30 mins and then allowed to warm to room temperature and stirred for 16 hours. The solvent was then removed *in vacuo* and the product purified by column chromatography on a Biotage Selekt with an Sfär cartridge (5g, silica – 60 μm) (2-20% methanol in DCM) to give (**5**) as a white film (19 mg, 32%). ^{1}H NMR (500MHz, MeOD) δ_{ppm} ^{1}H NMR (500 MHz, MeOD) δ 7.07 – 8.24 (10H, m, 8 x Ar<u>H</u> + 2 x N<u>H</u>), 4.57 (4H, dd, *J* = 4.0, 2.5 Hz, CHCC<u>H</u>$_2$OCO), 4.10 – 4.25 (4H, m, COC(CH$_3$)C<u>H</u>$_2$OCO), 3.52 (4H, tt, *J* = 8.0, 4.2 Hz, CONHC<u>H</u>$_2$CH$_2$CO), 2.80 – 2.87 (1H, m, C<u>H</u>CCH$_2$), 2.48 – 2.61 (4H, m, CONHCH$_2$C<u>H</u>$_2$CO), 1.11 – 1.15 (3H, m, C<u>H</u>$_3$). ^{13}C NMR (125MHz, MeOD) δ_{ppm} 173.4 (O<u>C</u>=OC(CH$_3$)), 172.8 (O<u>C</u>=OCH$_2$), 170.3 (NH<u>C</u>=O), 138.0 (Ar<u>C</u>H), 135.5 (Ar<u>C</u>), 132.7 (Ar<u>C</u>H), 129.6 (Ar<u>C</u>H), 128.2 (Ar<u>C</u>H), 78.4 (CH<u>C</u>CH$_2$), 76.7 (<u>C</u>HCCH$_2$), 66.5 (C(CH$_3$)<u>C</u>H$_2$C=O), 53.5 (CH3C<u>C</u>H$_2$OC=O), 47.6 (C=O<u>C</u>(CH$_3$)), 36.9 (<u>C</u>H$_2$NHC=O), 34.8 (OC=O<u>C</u>H$_2$CH$_2$NH), 18.0 (<u>C</u>H$_3$). MS *m/z* (ES$^+$): [M+Na]$^+$ calcd. for $C_{28}H_{32}B_2N_2O_{12}Na^+$, 633.2034; found 633.2031.

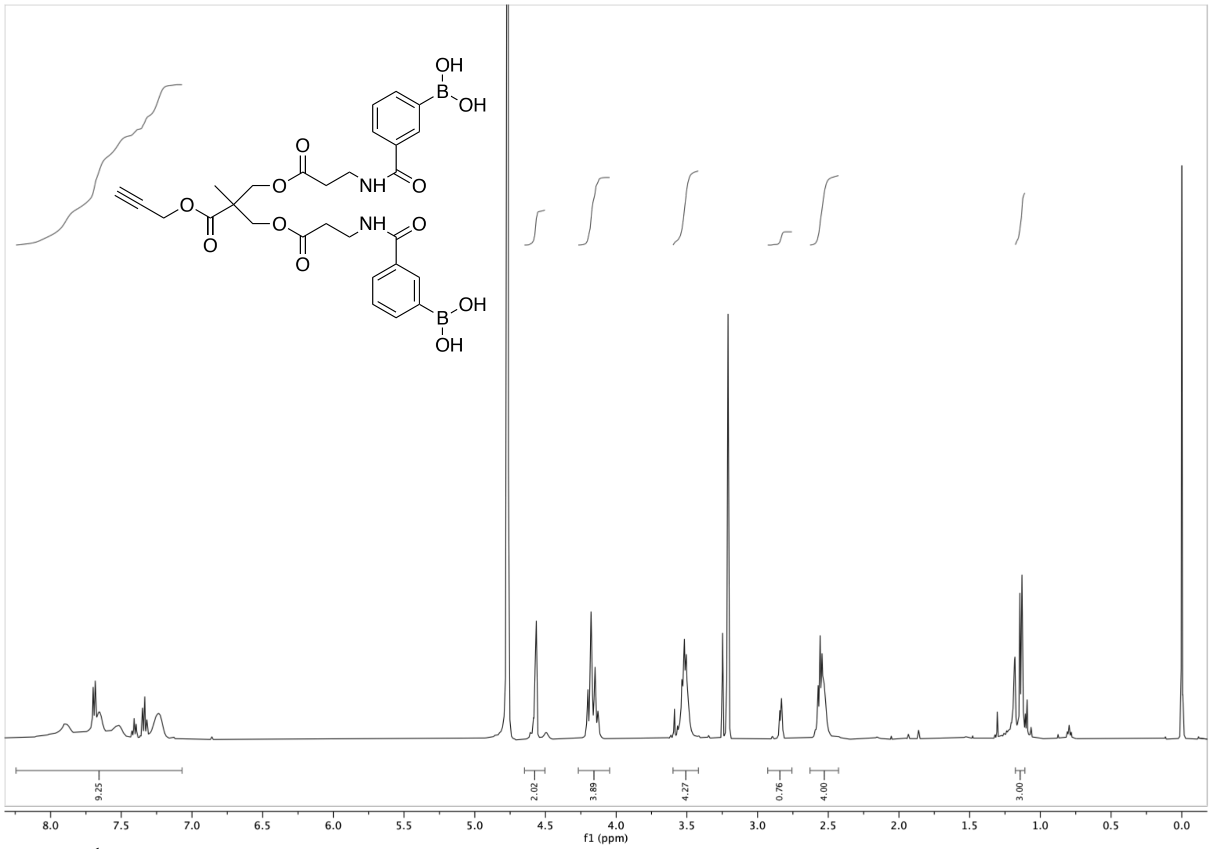

Fig. S5. ^{1}H NMR spectrum of B2-alkyne (**5**)

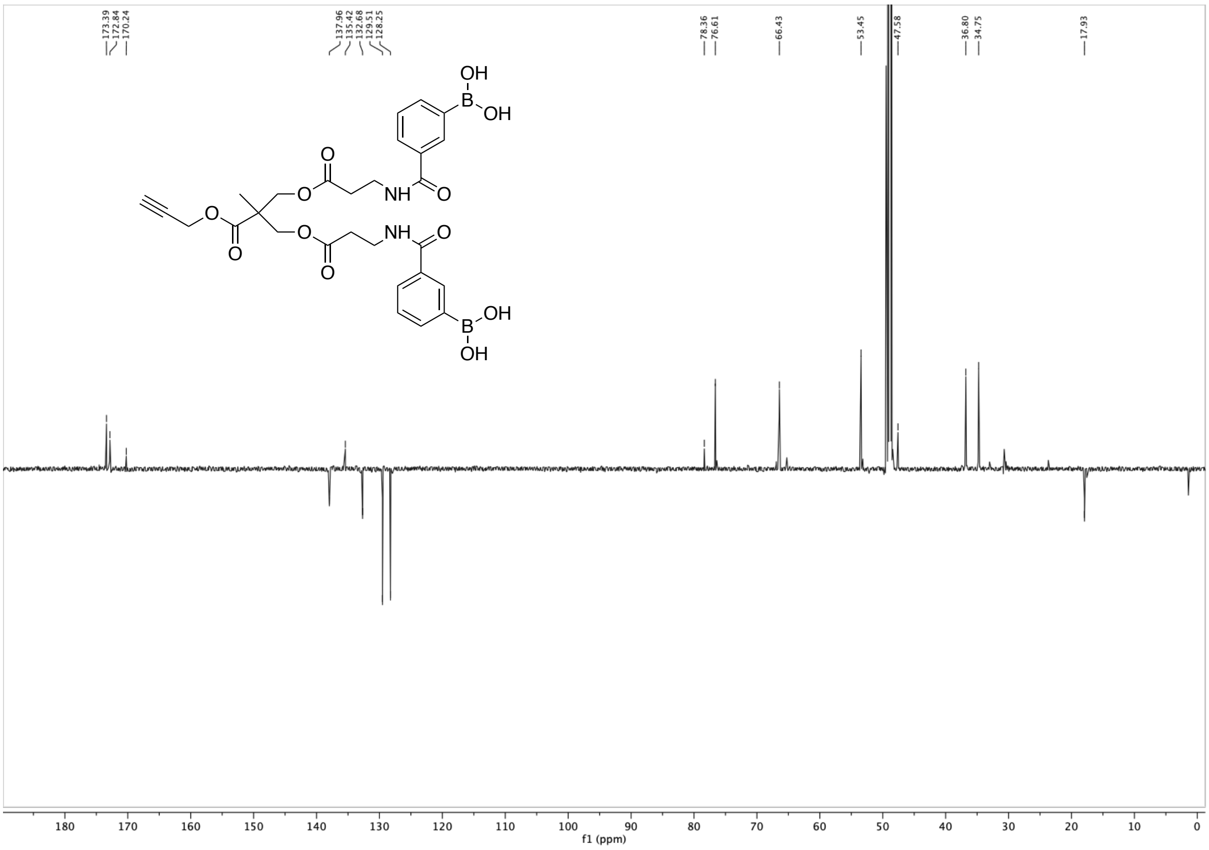

Fig. S6. ^{13}C NMR spectrum of B2-alkyne (**5**)

Polyester bis-MPA dendron (2 phenyl boronic acid, 1 azide(core), generation 1) (8)

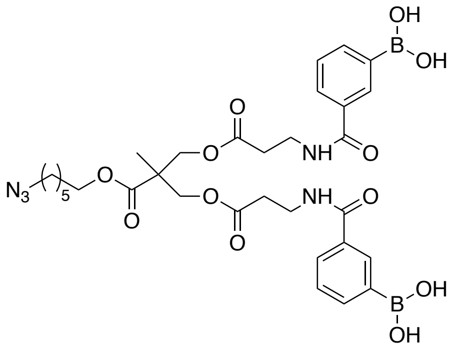

Polyester bis-MPA dendron (2 NHBoc, 1 azide (core), generation 1) (**6**) (50 mg, 0.083 mmol) was dissolved in trifluoroacetic acid (100 μL) and left at room temperature for 3 hours. The reaction was then concentrated *in vacuo* to give the deprotected amine (**7**) as a TFA salt. In a separate flask, 3-carboxyphenylboronic acid (41 mg, 0.249 mmol) was dissolved in anhydrous THF (3 mL) under nitrogen and cooled to 0 °C. DMF (3.9 μL, 0.050 mmol) was then added, followed by the dropwise addition of oxalyl chloride (51 μL, 0.598 mmol). The reaction mixture was then allowed to warm to room temperature and stirred for two hours before removing the solvent *in vacuo* to give crude 3-(chlorocarbonyl)phenyl boronic acid (**2**). The resulting residue was dissolved in anhydrous DCM (2 mL), under nitrogen, cooled to 0 °C. A mixture of the deprotected TFA salt (**7**) (0.083 mmol) and triethylamine (69 μL, 0.498 mmol) in anhydrous DCM (1 mL) was then added dropwise and the resulting mixture was stirred at 0 °C for 30 mins and then allowed to warm to room temperature and stirred for 16 hours. The solvent was then removed *in vacuo* and the product purified by column chromatography (19:1 EtOAc/iPrOH) to give (**8**) as a white film (17 mg, 30%). ^{1}H NMR (500MHz, MeOD) δ_{ppm} 7.25 – 8.12 (10H, m, 8 x Ar<u>H</u> + 2 x N<u>H</u>), 4.18 – 4.40 (4H, m, COC(CH_3)C<u>H</u>$_2$OCO), 3.95 – 4.16 (2H, m, CH_2C<u>H</u>$_2$OCO), 3.64 (4H, t, *J* = 7.0 Hz, CONHC<u>H</u>$_2$$CH_2$CO), 3.23 – 3.32 (2H, m, N_3C<u>H</u>$_2$$CH_2$), 2.66 (4H, t, *J* = 7.0 Hz, CONHCH_2C<u>H</u>$_2$CO), 1.01 – 1.68 (11H, m, 4 x CH_2C<u>H</u>$_2$$CH_2$ + C<u>H</u>$_3$). ^{13}C NMR (125MHz, MeOD) δ_{ppm} 173.0 (O<u>C</u>=OC(CH_3)), 171.5 (O<u>C</u>=OCH_2), 169.7 (NH<u>C</u>=O), 136.6 (Ar<u>C</u>H), 134.6 (Ar<u>C</u>H), 132.9 (Ar<u>C</u>), 131.8 (Ar<u>C</u>H), 127.0 (Ar<u>C</u>H), 65.3 (C(CH_3)<u>C</u>H$_2$C=O), 64.9 (CH_2<u>C</u>H$_2$OC=O), 50.9 (N3<u>C</u>H$_2$), 46.3 (C=O<u>C</u>(CH_3)), 35.5 (<u>C</u>H$_2$NHC=O), 33.4 (OC=O<u>C</u>H$_2$$CH_2$NH), 28.4 ($CH_2$<u>C</u>H$_2$$CH_2$), 28.1 ($CH_2$<u>C</u>H$_2$$CH_2$), 27.5 ($CH_2$<u>C</u>H$_2$$CH_2$), 25.1($CH_2$<u>C</u>H$_2$ CH_2), 16.7 (<u>C</u>H$_3$) MS *m/z* (ES$^+$): [M+Na]$^+$ calcd. for $C_{31}H_{41}B_2N_5O_{12}Na^+$ 720.2830; found 702.2830.

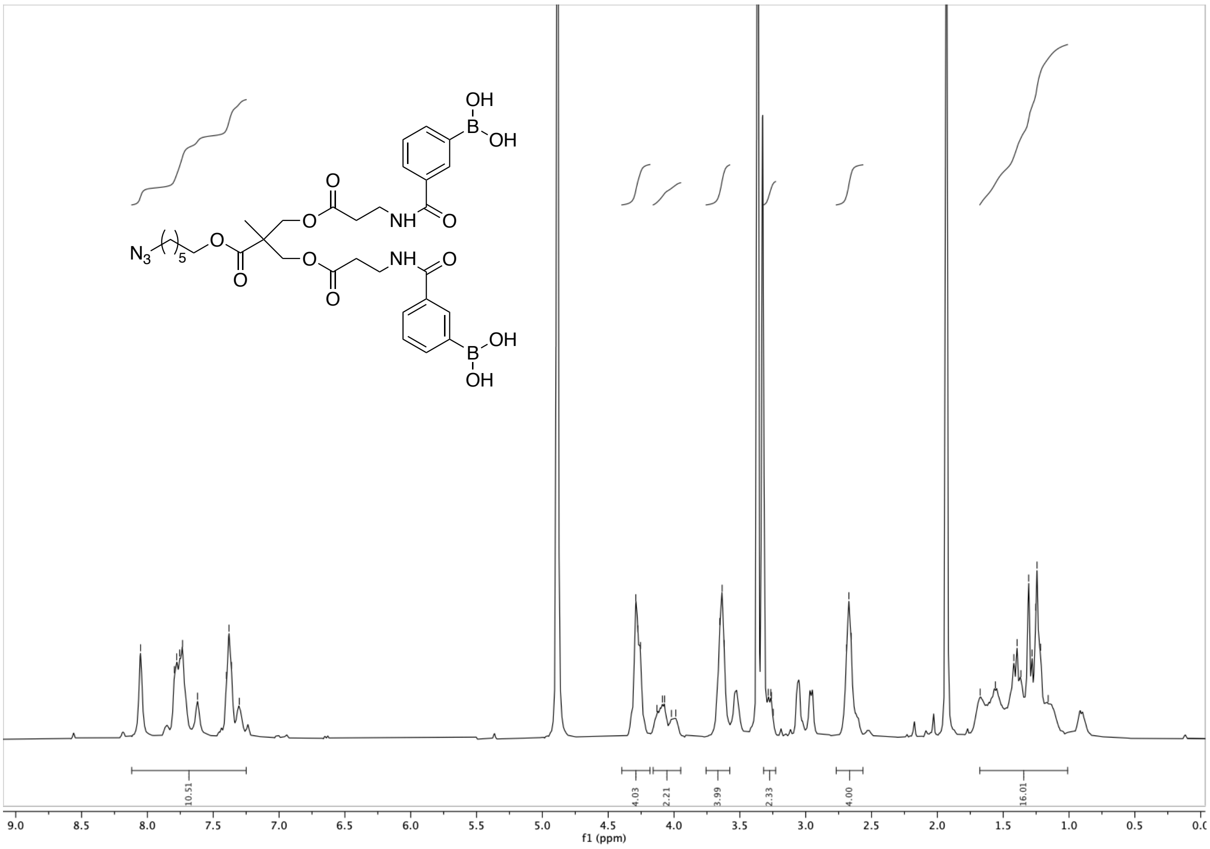

Fig. S7. ^{1}H NMR spectrum of B2-N_3 (**8**)

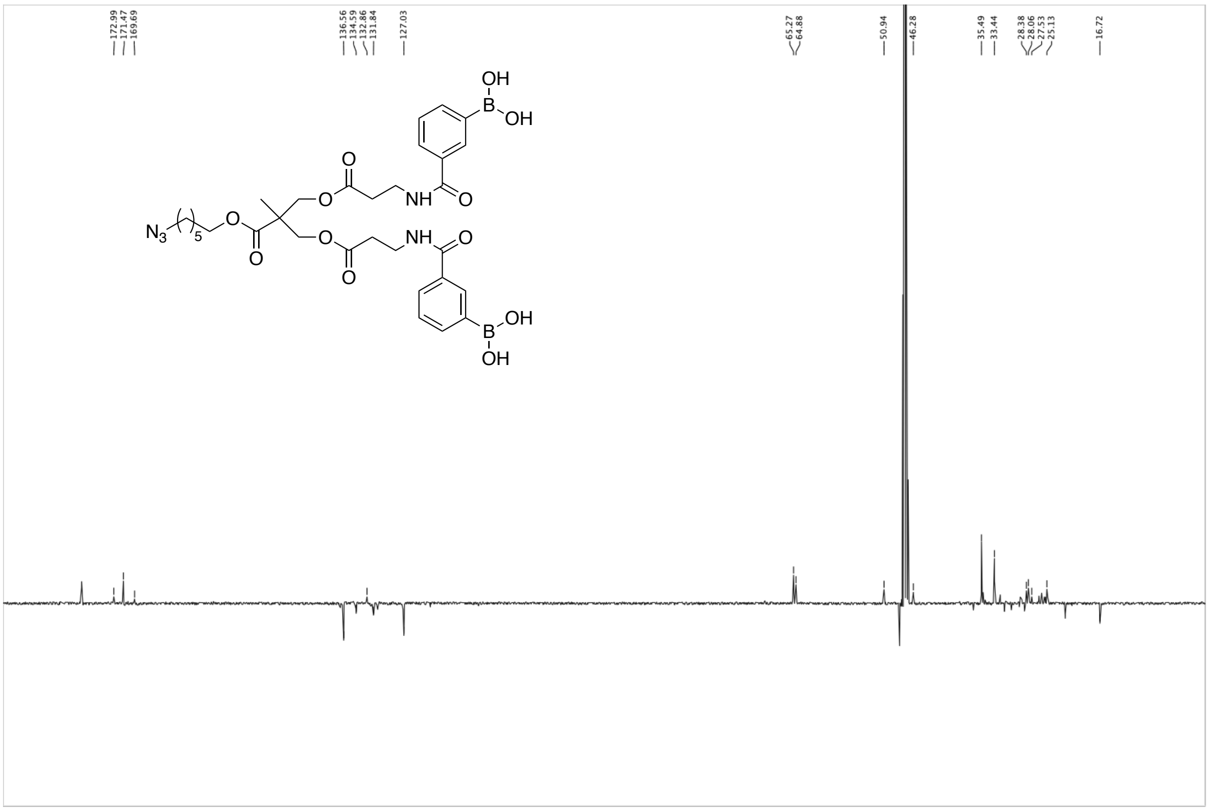

Fig. S8. ^{13}C NMR spectrum of B2-N_3 (**8**)

Supplementary Biological protocols

Bacterial strains, culture conditions and chemicals

Mycobacterium smegmatis MC^2155 (ATCC-700084) was routinely grown at 37 °C in Middlebrook 7H9 broth (BD Difco) supplemented with 0.2 % glycerol and 0.05 % Tween 80 or Tryptic Soy Broth (TSB) supplemented with 0.05 % Tween 80 or on Luria-Bertani (LB) agar. PBST is phosphate buffered saline supplemented with 0.05% Tween 80. All chemicals were purchased from Sigma-Aldrich unless otherwise stated. CalFluor Azide 488 was purchased from Click Chemistry Tools. The DBCO-Cy3 stock solution was prepared in DMSO at 5 mM. The CalFluor Azide 488 stock was prepared in H_2O at 1 mM. B2-alkyne stock was prepared at 50 mM in DMSO and B2-N_3 stock solution was prepared at 12.5 mM in 62.5% DMSO.

Determination of minimum inhibitory concentrations

The minimum inhibitory concentrations (MIC) of all compounds were determined using the resazurin reduction microplate assay (REMA) as described previously.[2] *M. smegmatis,* was grown to mid-log phase and the inoculum standardized to 1 x 10^6 colony forming units (CFU)/mL before addition to the prepared 96-well flat-bottom microtiter plate with 2-fold serial dilutions of each drug in media. A rifampicin antibiotic control was also added to each plate and the last column of the plate was used as a control without the addition of compound. The plates were incubated for 24 h before addition of resazurin (25 µL, one tablet of resazurin (VWR) dissolved in PBS (30 mL)). Following a further incubation for 24 h at 37 °C the plates were assessed for colour development. The MIC values were determined as the lowest concentration of drug that prevented the colour change of resazurin (blue is no bacterial growth) to resofurin (pink is bacterial growth).

Fluorescence analysis of B2-Alkyne and CalFluor Azide 488

In a black half area flat bottomed 96 well plate, B2-Alkyne (final concentration 1 mM) was added to PBST containing CalFluor Azide 488 (10 µM), $CuSO_4$ (50 µM), bovine serum albumin (BSA) (0.2 mg/mL), tris[(1-benzyl-1*H*-1,2,3-triazol-4-yl)methyl]amine (100 µM) and sodium ascorbate (2.5 mM) (final volume 50 µL) and incubated at room temperature with shaking, in the dark for 45 min. Controls of PBST alone, B2-Alkyne (1 mM) in PBST, CalFluor Azide 488 (10 µM) in PBST and CalFluor Azide 488 (10 µM), $CuSO_4$ (50 µM), bovine serum albumin (BSA) (0.2 mg/mL), tris[(1-benzyl-1*H*-1,2,3-triazol-4-yl)methyl]amine (100 µM) and sodium ascorbate (2.5 mM) in PBST were also prepared. Fluorescence was assessed at λ_{ex} of 488 nm and λ_{em} of 495-630 nm (Tecan Infinite M200, gain 60).

Fluorescence analysis of B2-N_3 and DBCO-Cy3

In a black half area flat bottomed 96 well plate, B2-N_3 (final concentration 100 µM) was added to PBST containing DBCO-Cy3 (100 µM) and incubated at room temperature with shaking, in the dark for 45 min. Controls of PBST alone, B2-N_3 (100 µM) in PBST, and DBCO-Cy3 (100 µM) in PBST were also

prepared. Fluorescence was assessed at λ_{ex} of 514 nm and λ_{em} of 538-680 nm (Tecan Infinite M200, gain 65).

Labelling of *Mycobacterium smegmatis* with B2-Alkyne and CalFluor Azide 488 dye for microscopy

M. smegmatis cells were cultured to an optical density (OD_{600}) of 0.8 in 7H9 media and incubated with **B2-alkyne** (1 mM), or DMSO (as a control), for 15 mins in Eppendorf tubes with shaking at room temperature. The cells were centrifuged (2,200 g, 5 min, 4 °C), resuspended in 7H9 media containing 2 mM phenylboronic acid and incubated at room temperature with shaking for 15 mins. The cells were centrifuged (2,200 g, 5 min, 4 °C) and fixed by resuspending in 4% paraformaldehyde at room temperature, with shaking for 15 mins. The cells were then centrifuged (2,200 g, 5 min, 4 °C), resuspended in PBST containing CalFluor Azide 488 (10 µM), $CuSO_4$ (50 µM), bovine serum albumin (BSA) (0.2 mg/mL), tris[(1-benzyl-1*H*-1,2,3-triazol-4-yl)methyl]amine (100 µM) and sodium ascorbate (2.5 mM) and incubated at room temperature with shaking, in the dark for 45 min. Control cells resuspended in PBST with no dye were also prepared. Cells were then pelleted (2,200 g, 5 min, 4 °C) and resuspended in 7H9 media containing 4 drops/mL of NucBlue live 4′,6-diamidino-2-phenylindole (DAPI) stain, incubated at room temperature in the dark with shaking for 20 mins, centrifuged (2,200 g, 5 min, 4 °C), washed once (PBST) and resuspended in PBST.

Labelling of *Mycobacterium smegmatis* with B2-Alkyne and CalFluor Azide 488 dye for flow cytometry

M. smegmatis cells were cultured to an OD_{600} of 0.8 in 7H9 media and incubated with **B2-alkyne** (1 mM), or DMSO (as a control), for 60 mins in Eppendorf tubes with shaking at room temperature. The cells were centrifuged (2,200 g, 5 min, 4 °C) and washed three times with PBST. The cells were then resuspended in PBST containing CalFluor Azide 488 (10 µM), $CuSO_4$ (50 µM), bovine serum albumin (BSA) (0.2 mg/mL), tris[(1-benzyl-1*H*-1,2,3-triazol-4-yl)methyl]amine (100 µM) and sodium ascorbate (2.5 mM) and incubated at room temperature with shaking, in the dark for 45 min. Control cells resuspended in PBST with no dye were also prepared. Cells were then pelleted (2,200 g, 5 min, 4 °C) and resuspended in PBST for flow cytometry analysis.

Labelling of *Mycobacterium smegmatis* with B2-N_3 and DBCO-Cy3 dye for microscopy and flow cytometry

M. smegmatis cells were cultured to an OD_{600} of 0.8 in 7H9 media and then incubated with **B2-N_3** (final conc 50 µM or 100 µM), or DMSO (as a control), for 30 min in Eppendorf tubes, with shaking at room temperature. The cells were then centrifuged (2,200 g, 5 min, 4 °C) and washed three times with PBST. The cells were resuspended in complete 7H9 media, the DBCO-Cy3 dye (final conc 50 µM or 100 µM) or DMSO (final conc 1% as a control) added and the cells incubated at room temperature, in the dark with shaking for 45 min then centrifuged (2, 200 g, 5 min, 4 °C) and washed four times with PBST.

Control cells resuspended in PBST with no dye were also prepared. Cells for microscopy were resuspended in 7H9 media containing 4 drops/mL of NucBlue live DAPI stain, incubated at room temperature in the dark with shaking for 20 mins, centrifuged (2,200 g, 5 mins, 4 °C), washed once (PBST) and resuspended in PBST. Cells for flow cytometry were resuspended in PBST for flow cytometry analysis.

Confocal microscopy

The labelled mycobacteria were spotted onto glass slides that had been prepared with 7H10 media (Middlebrook) containing 1.5% agarose and allowed to air-dry. Cover slips were placed over the sample which was fixed with an adhesive. Microscopy was performed on an LSM 880 confocal microscope (Carl ZEISS) using a Plan-Apochromat 100x/1.40 oil immersion objective lens and photomultiplier detectors (GaAsP, multialkali and BiG.2) or an Airyscan detector. Samples stained with DAPI and DBCO-Cy3 were excited with a 405 nm laser and 514 nm argon laser (2%), for the respective dyes, and detected within the 410–531 nm and 538–680 nm ranges. Samples stained with DAPI and CalFluor azide 488 were excited with a 405 nm laser and 488 nm argon laser (2%), for the respective dyes, and detected within the 410–495 nm and 495 – 630 nm ranges. Transmitted light was detected by the T-PMT detector. All images collected using PMT detectors were taken at x0 and x2 zoom. For Airyscan imaging, the same settings were used but with a zoom of x2, x4 or x6. Images were processed on ZEN 2.3 SP1 FP1, version 14.0.9.201. Imaging data are representative of at least three independent experiments.

Flow Cytometry

Flow cytometry of **B2-alkyne** samples labelled with CalFluor azide 488 was performed on a BD Influx™ cell sorter (BD Biosciences) running BD FACS Sortware™ software and equipped with 355-, 488-, 561-, and 642-nm lasers, detecting up to 24 parameters (21 fluorescence channels, two forward scatter channels and one side scatter). Samples were excited with a 488 nm excitation laser and detected with a 530/40 nm filter. 50,000 events recorded. A 100 µm nozzle was fitted, operating at a pressure of 20 psi (sheath) and 21.5 psi (sample). Stream and laser alignment was performed using BD Sphero™ Rainbow Calibration Particles (8 Peaks 3.0-3.4 µm). Voltage settings applied ensured that untreated control cells appeared at low emission intensities and all treated cells were within the detection range. Flow cytometry of **B2-N$_3$** samples labelled with DBCO-Cy3 was performed on a BD Accuri C6™ equipped with a 488 nm laser and 585/40 filter and 50,000 events recorded. BD CSampler Plus software (v 1.0.34.1) was used for data collection and processing. All flow cytometry experiments were performed in triplicate. FlowJo X 10.0.7r2 (Tree Star, Ashland, USA) was used for all statistical analysis and plotting of flow cytometry data.

References

1. Duval, F., van Beek, T.A. & Zuilhof, H. Sensitive Thin-Layer Chromatography Detection of Boronic Acids Using Alizarin. *Synlett*, 1751-1754 (2012).
2. Palomino, J.C. et al. Resazurin microtiter assay plate: Simple and inexpensive method for detection of drug resistance in *Mycobacterium tuberculosis*. *Antimicrob Agents Chemother* **46**, 2720-2722 (2002).
